# The Impact of a Ballet and Modern Dance Performance on Heart Rate Variability in Collegiate Dancers

**DOI:** 10.3390/sports7010003

**Published:** 2018-12-21

**Authors:** Rohan Edmonds, Meaghan Wood, Patricia Fehling, Sarah DiPasquale

**Affiliations:** 1 Department of Exercise Science and Pre-Health Professions, Creighton University, Omaha, NE 68178, USA; 2 Department of Health and Human Physiological Sciences, Skidmore College, Saratoga Springs, NY 12866, USA; Meaghan.wood@my.rfums.org (M.W.); pfehling@skidmore.edu (P.F.); 3 Department of Dance, Skidmore College, Saratoga Springs, NY 12866, USA; sdipasqu@skidmore.edu

**Keywords:** cardiac autonomic function, dance preparedness, ballet performance, modern performance

## Abstract

Heart rate (HR) variability (HRV) is a useful tool for assessing cardiac autonomic function and identifying potential readiness to perform in athletic populations, but has yet to be investigated in dance populations. As such, HRV may be able to provide valuable insight into the preparedness of dancers and the demands of performance in a collegiate dance population. 29 female dancers were monitored leading up to and following a dance performance. Analysis of HRV focused on the square root of the mean squared differences of the successive RR intervals (RMSSD). A one-way ANOVA, with Bonferroni post-hoc, paired with magnitude-based-inferences (MBI) with effect sizes (ES) were used to analyze changes during the Winter Dance Concert, while the Recovery-Stress Questionnaire for Athletes (REST-Q Sport) measured the frequency of stress of dancers. When compared to baseline (69.8 ± 1.7 bpm), mean (HR) was increased at both pre-show recordings (76.5 ± 2.1 bpm and 75.6 ± 1.8 bpm). In contrast, RMSSD was significantly diminished (*p* < 0.05) at both pre-show recordings (40.6 ± 28.4 ms and 40.5 ± 21.8 ms) as compared to baseline (70.3 ± 38.4 ms). Dancers reported increased (*p* < 0.05) self-efficacy before the second show and at 36 h post-concert. As expected, Dance Exposure (DE) increased significantly (*p* < 0.05), while Academic Exposure (AE) was similar, during the week leading up to the dance concert. The results suggest dancers responded to concert dance performances similarly to other athletic populations approaching intense competition by exhibiting decreased parasympathetic activity prior to the dance performances, which returned to baseline values 36 h after their performances. Given the increase in self-efficacy, these fluctuations may indicate a readiness to a performance comparable to athletes.

## 1. Introduction

Dance is considered both an art and sport, demanding the ability to express emotion while performing physically demanding movements that require stamina, strength, and flexibility [1,2]. Dancers train and rehearse leading up to high-pressure performances, which can elicit emotions, such as arousal or fear [3]. Appropriate fitness for dancers depends upon the individual’s ability to work under aerobic and anaerobic conditions and develop elevated levels of muscle tension and joint mobility while maintaining body composition that is aesthetically appropriate [1,4]. Multiple studies have suggested that the aerobic demands of dance performance are not met through training and rehearsal, which could greatly inhibit performance quality in ballet and modern dancers [1,4,5,6]. Stress and anxiety are understood aspects of competitive sports [7,8], and similarly, there is stress placed on dancers during high pressure performances [3]. 

While dance was once considered a form of visual art, a new recognition and understanding of the physical demands of dance and its similarities to athletics is widely accepted [9]. In a collegiate setting, dancers may work towards culminating performances toward the end of an academic term, unlike professional dancers who perform on a regular basis. Therefore, the training regimen of collegiate dancers must be assessed separately from that of the professional dancer to account for the unique performance schedule of this population. Furthermore, in a collegiate setting, dance students may participate in both academic and dance training every day, which is similar to the routine of a collegiate athlete. While dancers are widely accepted as athletes [9], a lack of understanding of collegiate dance performance readiness exists.

Heart rate (HR) variability (HRV) is a convenient and non-invasive tool that is used in athletic populations to quantify cardiac autonomic influences on the heart by measuring the variance in RR intervals [10]. The autonomic nervous system (ANS) is responsible for conducting neural activity that controls the sympathetic (SNS) and parasympathetic nervous systems (PNS) [11]. In healthy individuals at rest, variability exists between heartbeats due to fluctuating innervations from the SNS and PNS [11].

An acute decrease in HRV approaching athletic competition has been shown to elicit athletic preparedness [12,13,14]. However, a prolonged decrease (>72 h) in HRV post-competition might result in decreased athletic performance [10]. While prior research has found HRV to be a useful tool for monitoring athletic populations approaching intensive competition [12,13,14], it has yet to be utilized within a dance population in the same capacity [15,16]. As dancers complete extensive practice through class and rehearsals to prepare for dance performance (comparable to training within an athletic population), HRV may provide valuable insight into the physiological demands of dance.

As such, the purpose of the current study was to examine acute fluctuations in cardiac autonomic function in a cohort of collegiate dancers leading up (one week prior) to an intensive modern and ballet concert weekend. It was hypothesized that, as the cardiorespiratory demands of the dancers were increased due to an intensive weekend of performances, HRV would be lower when compared following the concert when compared to baseline values. This research may have implications on determining whether collegiate dancers are adequately prepared for the demands of performance or are fatigued by performance intensity.

## 2. Materials and Methods

### 2.1. Participants 

The participants of this study were students from a liberal arts college in the United States. The 29 female dancers enrolled in this study were on average 20.0 ± 1.1 years old, weighed 58.9 ± 5.3 kg, and were 165.5 ± 5.8 cm tall. Participants were declared, or intended to declare, dance majors (n = 19) or dance minors (n = 10). Dancers were asked to indicate their concentrations of dance training and they were allowed to indicate multiple concentrations; 20 participants stated modern dance, 17 participants stated ballet dance, and two participants stated tap dance. Inclusion criteria were enrollment in more than six credit hours of modern and ballet dance technique courses per week, and selection via audition to dance in at least one faculty-led workshop that would be performed in small groups as part of the Winter Dance Concert in December 2016. Exclusion criteria included having a BMI indicating a classification of overweight or obese or if any serious medical condition or soft tissue injury was revealed in the completed medical history questionnaire. No participants were excluded because of these criteria. Participants signed an informed consent form prior to all testing and they were compensated for their enrollment with a $10 Starbucks gift card. This study received approval from the college Institutional Review Board. 

### 2.2. Procedures

**Figure 1:**
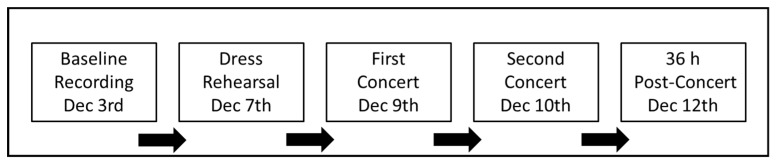
A timeline of heart rate (HR) data collections (N = 29).

Participants’ HR (BioHarness, Zephyr Technology, Annapolis, MD, USA) were recorded at baseline (between 8–9 am), the morning of dress rehearsal (between 8–9 am), 30-minute prior to warm-up class for both concert performances (between 6–7 pm), and the Monday morning after performance weekend (between 8–9 am) (Figure 1). All HR recordings were 10 minutes in length and were taken in an upright seated position. HRV was analyzed during the most stable (visually inspected for a variance of less than 10 bpm) five-minute period within the 10-minute recording and participants were asked to sit quietly and breathe normally. Analysis of HRV focused solely on mean HR and the square root of the mean squared differences of the successive RR intervals (RMSSD) as a well-documented measure of cardiac vagal modulation [10,13,17].

### 2.3. Descriptive Variables

At each HR recording, participants completed the Recovery-Stress Questionnaire for Athletes (RESTQ-Sport) to measure the frequency of current stress and recovery-associated activities [18]. This 53-question survey describes the mental, emotional, and physical well-being of athletes [18]. The questionnaire operates on a scale ranging from 0 (never) to 6 (always), indicating how often the respondent participated in various activities or experience relevant states. Participants completed a basic health questionnaire prior to each HR data recording session to record the number of hours that the participant slept the night before data collection. The stage of menstrual cycle was not controlled for, as previous research has shown no changes in cardiac autonomic function associated with the phases of the menstrual cycle [19].

The variables dance exposure (DE) [20] and academic exposure (AE) were recorded as descriptive variables to determine any additional contribution of stress or fatigue for this collegiate population. Participants recorded AE for a week at baseline the week prior to performance, and during performance week. Participants kept a daily written log of the hours spent in academic classes, studying, or completing assignments, and reported this value during HR collection sessions. To track DE, participants completed a daily self-report of the number of hours that were spent dancing during a typical week of dance class, and for the week of the Winter Dance Concert. Participants recorded DE through the following categories: ballet, modern, and tap technique classes, faculty-led rehearsals, student dance clubs, student-led rehearsals, independent study, improvisation, and performance. Before their first HR recording, the participants filled out an intake form to gain information on their dance background, injury prevention education, and injury history. 

### 2.4. Analysis

All RR interval recordings were analyzed via Kubios HRV software (v2.2, University of Kuopio, Finland). Any identified artifact was corrected using Kubios’ in built piecewise cubic spline interpolation [17]. Using the Statistical Package for Social Sciences (SPSS) software (v21, SPSS INC, Chicago, IL, USA), a one-way analysis of variance, with a Bonferroni post-hoc, was utilized to examine the differences over time for REST-Q Sport, DE, AE, Mean HR, and RMSSD data. All HRV data were log-transformed before analysis to reduce bias from non-uniformity, as HRV has been reported to lack a normal distribution [17]. Mean HR and RMSSD were analyzed using Magnitude-based inferences (MBI) to determine the practical significance of changes using standardized differences in means or Cohen’s *d* effect size (ES) [20]. The threshold values of ES were set to small (0.2), moderate (0.6), large (1.2), and very large (2.0) [21]. These MBI values were undertaken using the smallest worthwhile coefficient of variation (%SWC), calculated as 0.2 of between-subjects’ SD, with age being considered as a potential influencing factor [21]. In line with previous research, the chances of significant positive, trivial, or negative changes were assessed qualitatively: almost certainly not (<0.5%), very unlikely (0.5–5%), unlikely (5–25%), possibly (25–75%), likely (75–95%), very likely (95–99.5%), and almost certainly (99.5%) [21].

## 3. Results

### 3.1. Heart Rate Variability

**Figure 2:**
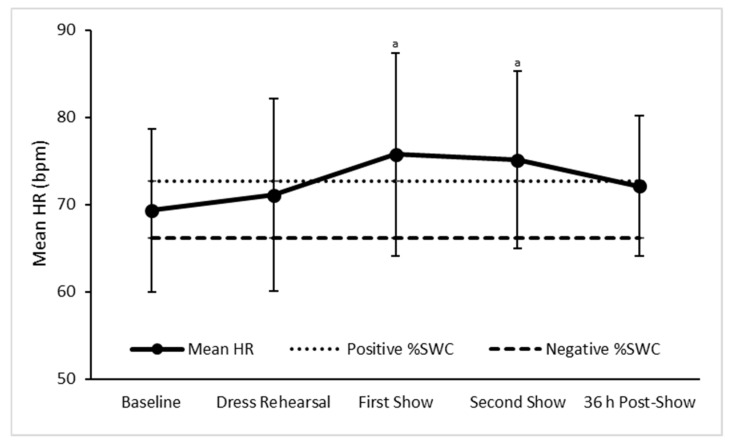
Mean ± SD heart rate of dancers at baseline, during performance week, and after performance. Positive %SWC—the smallest positive worthwhile change for group heart rate. Negative %SWC—the smallest negative worthwhile change for group heart rate. ^a^—Likely higher than baseline, *p* < 0.05 compared to baseline.

Mean HR was significantly higher at the first (*p* = 0.018) and second (*p* = 0.006) pre-concert recordings as compared to baseline, while similar at all other time points (Figure 2). Additionally, when determining the practical significance of change, mean HR was likely to be higher at the first (92/8/0, ES = 0.36) and second (93/7/0, ES = 0.33) pre-concert recordings when compared to baseline (Figure 2, Table 1). All other changes in mean HR were trivial (Figure 2, Table 1). 

**Figure 3:**
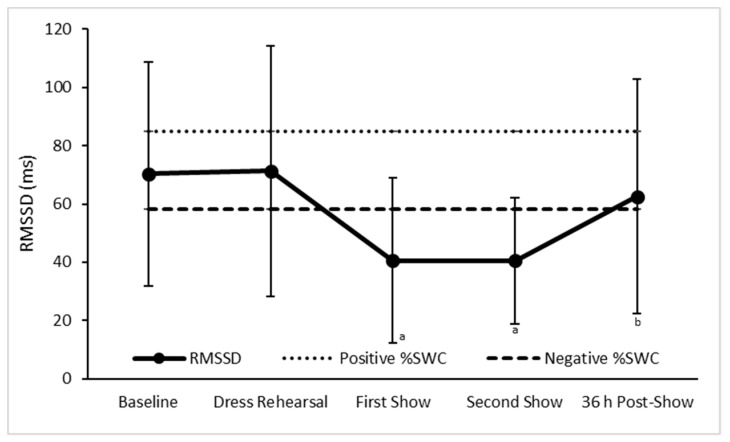
Mean ± SD square root of the mean squared differences of the successive RR intervals (RMSSD) of dancers at baseline, pre-concert, and post-concert. Positive %SWC—the smallest positive worthwhile change for group RMSSD. Negative %SWC—the smallest negative worthwhile change for group RMSSD. ^a^—Most likely lower than baseline, *p* < 0.05 compared to baseline. ^b^—*p* < 0.05 compared to the second show

In contrast, RMSSD was significantly lower at the first (*p* = 0.002) and second (*p* < 0.001) pre-concert recordings when compared to baseline and the dress rehearsal (Figure 3), while the second pre-concert recording was also significantly lower (*p* = 0.032) as compared to 36hr post-show (Figure 3). Furthermore, when determining the practical significance of change, RMSSD was most likely lower at the first (0/0/100, ES = −0.66) and second (0/0/100, ES = −0.61) pre-concert recordings when compared to baseline (Figure 3, Table 1). 

### 3.2. Rest-Q Sport

**Figure 4:**
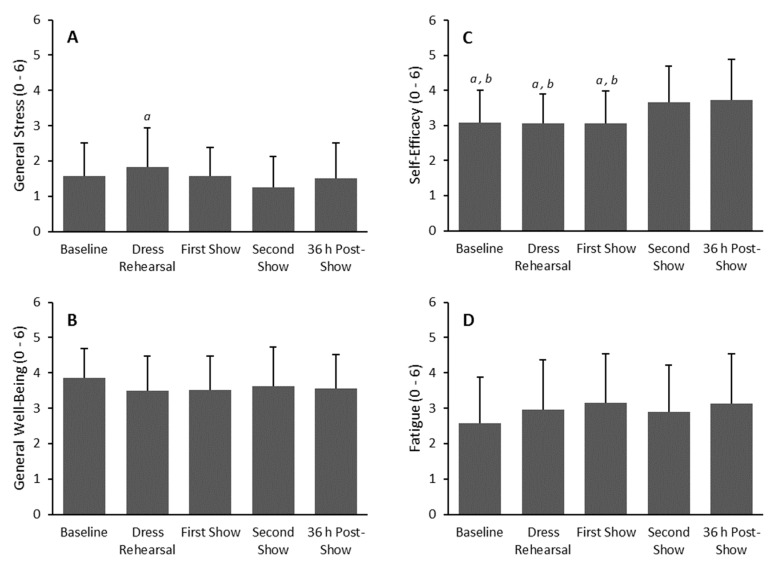
Mean ± SD of general stress (**A**), general well-being (**B**), self-efficacy (**C**), and fatigue (**D**) of dancers at baseline, dress rehearsal, before the first and second show, and 36 h post-show (N = 29), *^a^ p* < 0.05 vs. second show. ^b^ *p* < 0.05 vs. 36 h post-show.

Self-efficacy was significantly higher (*p* < 0.05) prior to the second performance and 36 hours post-concert (Figure 4). General stress was also the highest at the pre-dress rehearsal recording, however, it was only significantly higher when compared to the second concert performance (Figure 4). 

### 3.3. Dance and Academic Exposure

During the performance week, DE was 19.6 ± 7.3 h, significantly higher (*p* < 0.05) than the baseline week (14.5 ± 5.8 h). In contrast, AE was similar during the performance week (32.7 ± 12.2 h) and the baseline week (35.7 ± 11.4 h).

## 4. Discussion

The purpose of the current study was to examine the impact of a dance concert weekend on cardiac autonomic function in collegiate dancers. To our knowledge, the current study was the first of its kind to document a decrease in dancers preparing for dance performances. The major findings of this study were that HRV was significantly lower at both pre-concert recordings, with HRV returning to baseline at the post-concert weekend. Unlike the field of athletics, dance is inherently subjective, and while prior research has developed methods to objectively evaluate dance performance [22], these measures were not utilized in the current study. The inability to subjectively evaluate dance performance in the current study justified the use of a questionnaire to subjectively gauge how dancers felt about their performance, feelings of general well-being, fatigue, and stress over the monitoring period.

### 4.1. Arousal versus anxiety

A key finding of the current study was the lowered cardiac PNS activity found at both pre-concert recordings. Coupled with an increased HR, the finding indicated increased cardiac SNS modulation. Prior investigations have reported similar trends in HRV obtained from elite athletic populations [12,13,14]. Acute decreases in HRV leading up to athletic competition were indicative of a readiness to perform (arousal) [13] or a potential anxiety response [12,14]. The reported increase in self-efficacy after each show, and the lack of increase in stress prior to each show, suggests that the dancers were aroused, rather than anxious, leading up to their performances. Indeed, self-efficacy was defined by the RESTQ-Sport as denoting expectation and competence regarding optimal performance [18], further supporting this notion of a readiness to perform. 

Artistic performance in front of audiences has been found to elicit performance anxiety or stage fright, however it has been determined that trained dancer populations have displayed arousal rather than anxiety prior to high-pressure performances [23]. Prior research has been conducted to understand the possible correlation between stress and anxiety during dance performances, finding that modern dance performances were not more stressful and they did not require more effort when compared to rehearsal [3]. Additionally, the authors reported that the majority of modern dancers reported via questionnaire that their motive for dancing was arousal seeking [3]. In athletic populations, increased physiological arousal has been linked to excitement as opposed to anxiety [23]. High-level and skilled athletes have experienced increased arousal in a non-stressful and enjoyable manner as opposed to anxiety [12,13,14]. The anxiety of stage fright in musicians and actors was associated with a decreased ability to perform [24], while the arousal of athletes was associated with an increased ability to perform [23]. The findings of reported self-efficacy may indicate that the dancers were not anxious but aroused as the physiological demands placed on them may be more similar to those experienced by athletes rather than other artistic performers, like actors or musicians. The beneficial effects of increased HR and increased SNS activity may have enhanced the dancers’ readiness to perform leading into the dance concert.

### 4.2. Dancers as Athletes

With previous research highlighting the similarities between dancers and athletes [9], it is, potentially, not surprising that the dancers in the current study responded as such. Despite an increase in DE over the week leading up to the dance concert weekend, the dance cohort appeared to respond well to these elevated demands as HRV returned to baseline values within 36 h of the concert weekend. Similar responses have been documented in athletic populations following strenuous training [25] and they suggest that the dancers may be accustomed to these occasional increases in workload and stress, leading to a favorable cardiac autonomic response. Additionally, the increased feelings of self-efficacy support this belief that the dancers responded favorably to the increased workload. While previous research has suggested supplementary cardiorespiratory training is beneficial for dance students [1], care should be taken when manipulating the training loads of this population to ensure that the aesthetic content of dance is not negatively affected [9]. Indeed, the overall results of the current study suggest that the dancers were adequately prepared and they responded appropriately to the increased demands of the winter concert performances

### 4.3. Limitations

The self-reported nature of DE and AE contains inherent bias, and more objective methods of collection are recommended for future research. Additionally, sample sizes that are equal in sex distribution are recommended for future research. Unlike the field of athletics, dance is inherently subjective, and although prior research has developed methods to objectively evaluate dance performance [22], these measures were not utilized in the current study. It is recommended that future study designs incorporate an objective scoring method when assessing dance performance. Given the nature of the study, it was not possible to record HRV at the same time of day throughout the study; as such, the potential for diurnal variation may be a confounding variable. Furthermore, there were no objective measures of aerobic and/or anaerobic fitness utilized in the current study design, with statements about the dancer’s fitness pertaining to how many hours of dance that they completed each week. Additionally, no restrictions of strenuous exercise outside of dance were accounted for. Further research should aim to quantify a dance cohort’s fitness, while limiting any external source of strenuous exercise, to better document and evaluate fluctuations in cardiac autonomic balance in response to dance concerts. As in the current study, while HRV analysis has typically centered on the examination of RMSSD as a marker of cardiac vagal modulation, future research may benefit from analysis of additional HRV indices, such as the standard deviation of all normal to normal RR intervals (SDNN). The incorporation of supplementary HRV measures will only strengthen its application in the field. Lastly, while the use of MBI within HRV research is well documented, recent discussions have outlined the potential limitations that are associated with its use [26]. As such, inferential statistics has been used in the current study to provide additional support to the current findings of this research.

## 5. Conclusions

In acceptance of the hypothesis, dancers reported a reduction in HRV following a dance concert performance. Additionally, and comparable to athletic populations, the decreased cardiac vagal activity leading into the dance concert, paired with the increased self-efficacy, suggest that the dancers exhibited a readiness to perform and adequate preparedness for the Winter Dance Concert. Despite the increased DE reported during the lead up to the concert weekend, given that cardiac autonomic balance returned to baseline within two days after the show, it appears that the dancers responded well to the workload associated with a typical dance concert weekend by exhibiting a state of physiologic arousal and increases in self-efficacy. These findings support that dancers are training adequately to perform optimally while maintaining the ability to recover the weekend after their major performance of the semester. Given the intense workload that is placed on the dance population, it would be beneficial to examine the chronic training responses within this population. A long-term study is warranted to examine how dancers cope with the stressors of a dance semester, as it is known that extended periods of reduced PNS activity may be detrimental to performance [10].

## Figures and Tables

**Table 1:** Mean values + SD, variance from baseline, effect size (ES), and qualitative inferences for HRV.

Heart Rate Variability	Baseline	Dress Rehearsal	First Concert	Second Concert	36 h Post-Concert
Mean HR (bpm)	69.3 ± 9.4	71.1 ± 11.0	75.8 ± 11.6 *	75.1 ± 10.1 *	72.1 ± 8.0
%Δ (95% CI)	-	2.3 (−2.9, 7.9)	8.9 (3.2, 14.9)	8.3 (3.9, 13.0)	4.4 (−0.5, 9.4)
ES	-	0.09	0.35	0.33	0.18
QI	-	Trivial	Likely Positive	Likely Positive	Trivial
RMSSD (ms)	70.3 ± 38.4	71.3 ± 43.1	40.6 ± 28.4 *	40.5±21.8 *	62.5 ± 30.2
%Δ (95% CI)	-	−2.4 (−18.4, 16.9)	−44.5 (−56.2, −29.6)	−43.0 (−54.1, −29.3)	−16.1 (−30.1, 1.1)
ES	-	−0.02	−0.61	−0.58	−0.18
QI	-	Trivial	Most Likely Negative	Most Likely Negative	Trivial

HR—heart rate; bpm—beats per minute; %Δ—percentage change from baseline; CI—confidence interval; ES—effect size; QI—qualitative inference; RMSSD—square root of the mean squared differences of the successive RR intervals. * *p* < 0.05 compared to baseline.
